# Supplementary material for: eQTM (expression quantitative trait methylation) Atlas: a comprehensive resource of over 11 million DNA methylation–gene expression associations through across 11 tissues and 4 diseases
Source: bioRxiv. 2026 Jun 10:2026.06.07.730721. Preprint. [Version 1] doi: 10.64898/2026.06.07.730721 (PMC13278183; doi:10.64898/2026.06.07.730721)
Supplement: Supplementary file 1 [file NIHPP2026.06.07.730721v1-supplement-1.pdf]

bioRxiv preprint doi: https://doi.org/10.64898/2026.06.07.730721; this version posted June 10, 2026. The copyright holder for this preprint (which was not certified by peer review) is the author/funder, who has granted bioRxiv a license to display the preprint in perpetuity. It is made available under a CC-BY 4.0 International license.

Table S1. Number of cis and trans eQTM associations by cohort

Cohort	cis	trans
Colonomics	165	0
EVA-PR: All Subjects	23,953	9,054,589
EVA-PR: Atopic asthma + controls	9,108	2,131,500
EVA-PR: Atopic Asthma Only	1,485	101,871
EVA-PR: Controls	289	1,229
EVA-PR: White Blood Cell	10,385	146,659
EvoImmunoPop	811	0
Framingham Heart Study	10,000	0
FUSION	0	36,516
GTEx: BreastMammaryTissue	6,318	0
GTEx: ColonTransverse	6,650	0
GTEx: Lung	8,686	0
GTEx: MuscleSkeletal	4,319	0

bioRxiv preprint doi: https://doi.org/10.64898/2026.06.07.730721; this version posted June 10, 2026. The copyright holder for this preprint (which was not certified by peer review) is the author/funder, who has granted bioRxiv a license to display the preprint in perpetuity. It is made available under a CC-BY 4.0 International license.

Cohort	cis	trans
GTEx: Ovary	9,251	0
GTEx: Prostate	6,203	0
GTEx: Testis	2,439	0
GTEx: WholeBlood	3,916	0
HELIX: Whole Blood Autosomal	32,677	0

Supplementary Table 1 – Sequencing Technology Implemented Across Cohorts

Study/Cohort	Data Type	Technology Used
**EVA-PR**	RNA-seq	Illumina NextSeq 500 platform
**FUSION**	RNA-seq	Illumina HiSeq 2000 platform
**Framingham Heart Study**	RNA-seq	Illumina NovaSeq platform
**Colonomics**	RNA-seq	AB SOLiD System
**EvoImmunoPop**	RNA-seq	Illumina HiSeq 2000 platform
**HELIX**	RNA (extracted)	Tempus tubes; MagMAX kit; QC: 2100 Bioanalyzer and NanoDrop 1000; Infinium HumanMethylation450K BeadChip (Illumina)
**GTEx**	RNA-seq	Illumina TruSeq (non-stranded, polyA+ selection) platform

bioRxiv preprint doi: https://doi.org/10.64898/2026.06.07.730721; this version posted June 10, 2026. The copyright holder for this preprint (which was not certified by peer review) is the author/funder, who has granted bioRxiv a license to display the preprint in perpetuity. It is made available under a CC-BY 4.0 International license.

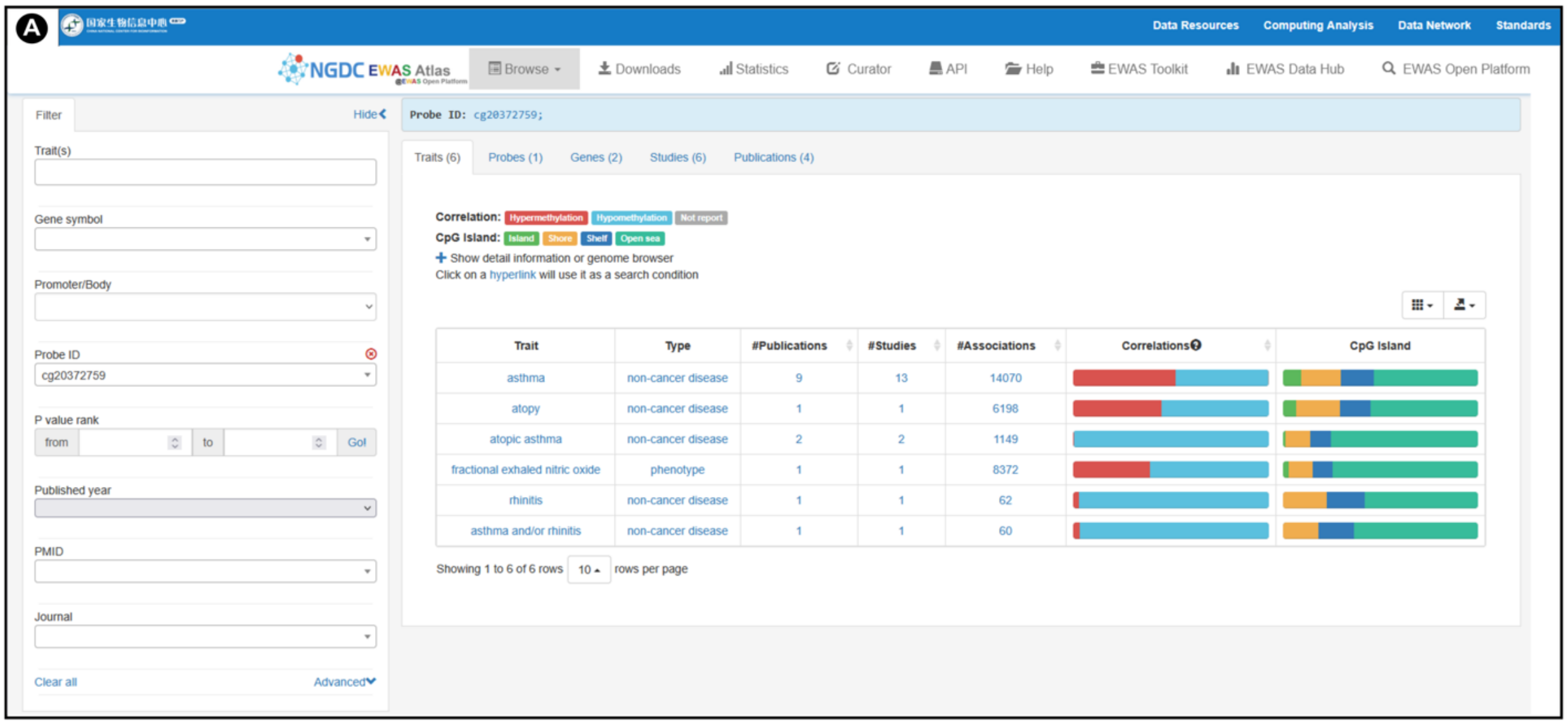

Figure S1. EWAS atlas results for cg20372759. Representative view from the EWAS Open Platform. Data is shown following selection of the "Open in EWAS atlas" box within the EWAS-eQTM Integration tab of Figure 3D.
